# Changes in regional homogeneity of the social brain in individuals with autism spectrum disorder after social skills training

**DOI:** 10.3389/fpsyt.2026.1674370

**Published:** 2026-03-13

**Authors:** Yu-lu Yang, Hui Wang, Siuching Kat, Zeng-Hui Ma, Ting-Ni Yin, Ya-Jing Sun, Xin-Zhou Tang, Xiao-Yun Gong, Duo Wang, Lei Li, Xue Li, Jing Liu

**Affiliations:** 1 Peking University Sixth Hospital, Peking University Institute of Mental Health, National Health Commission of the People's Republic of China (NHC) Key Laboratory of Mental Health (Peking University), Beijing, China; 2 Beijing Key Laboratory for Big Data Innovative Application of Child and Adolescent Mental Disorders, National Clinical Research Center for Mental Disorders (Peking University Sixth Hospital), Beijing, China; 3 Mental Health Center, National Center for Mental Disorders, West China Hospital, Sichuan University, Chengdu, China

**Keywords:** autism spectrum disorder, brain mechanism, regional homogeneity, social brain network, social skill training

## Abstract

**Introduction:**

This study examined changes in regional homogeneity (ReHo) following Social Skills Training (SST) and their association with improvements in social deficits in individuals with autism spectrum disorder (ASD).

**Methods:**

44 adolescents and adults with ASD (aged 12-30) were recruited, 38 participants (20 in training group, 18 in control group, matched for sex, age, and IQ) were retained after quality control of MRI data. The training group underwent magnetic resonance imaging (MRI) scans and assessments of Aberrant Behavior Checklist (ABC) and Social Responsiveness Scale (SRS) before and after a 14-week SST program, while the control group completed the same MRI scans and assessments at the same time point but did not receive SST.

**Results:**

Resting-state functional MRI analyses revealed significant group × condition interactions in five social brain regions, including the right medial frontal gyrus, right insula, and left medial superior frontal gyrus. At the endpoint of SST, the training group showed reduced ReHo in these regions alongside significant decreases in scores of ABC total, social withdrawal factor, SRS total, social awareness, social cognition, social communication factors. The control group, in contrast, showed only limited improvements on specific subscales, while the training group demonstrated a broader pattern of behavioral gains. We also found an exploratory association between the decrease in ReHo of the right medial frontal gyrus and the reduction in the ABC total score in training group.

**Discussion:**

These findings indicate that SST may modulate local functional connectivity within the social brain networks, and these connectivity changes may correlate with observed behavioral improvements.

## 1 Introduction

Autism Spectrum Disorder (ASD) is a neurodevelopmental disorder characterized by impairments in social interaction and communication, along with restricted and repetitive behaviors as well as limited interests (1). The prevalence of ASD has shown an increasing trend, reaching 2.8% among 8-year-old children in the United States (2), 1.77% among 16-year-old in the New York-New Jersey metropolitan area (3), and estimated 2.21% among adults aged 18 and older in the United States (4). ASD significantly affects the social functioning of individuals, resulting in a significant burden on families and society (5) and has become a public health issue of global concern.

Among core symptoms of ASD, social deficits are the most important symptoms. These deficits, including impaired social perception, deficits in social cognition, and challenges in relationship development and maintenance, emerge during early developmental stages and are marked by a fundamental absence of social motivation (6). All of these cause significant and profound hardships and challenges for affected individuals (7), resulting in individuals with ASD facing poor peer relationships, higher levels of loneliness, lower social status, and susceptibility to depression than their peers (8). ​Consequently, developing and using evidence-based interventions targeting social deficits is essential to face these challenges. To date, there are no specific and effective pharmacological treatments for social deficits of ASD. Long-term training remains the primary treatment approach for ASD (9). Recent studies have demonstrated that social skills training (SST), which encompasses the social skills interventions such as verbal and nonverbal communication, and facilitating social interactions (e.g., initiating and maintaining conversations) and problem-solving (addressing interpersonal conflicts and bullying) (10), can improve social impairments in individuals with ASD (11). It can effectively improve impairments in social interactions among individuals with ASD across various age groups (12, 13). ​​Our prior work​​ (14, 15) also demonstrated the effectiveness of SST in ameliorating social interaction impairments among children, adolescents, and adults with ASD. However, the neurobiological mechanisms underlying SST-induced improvements remain poorly characterized.

While ​​the precise neuropathological mechanisms underlying ASD remain incompletely elucidated​​, emerging evidence ​​suggests that aberrant local brain function may be a key contributor to ASD social deficits. Regional Homogeneity (ReHo) (16), a voxel-based measure of local neural coherence in resting-state functional magnetic resonance imaging (R-fMRI), provides a sensitive tool for characterizing aberrant local brain function in ASD. Studies consistently reveal ReHo abnormalities in ASD,​​ characterized by both hyperconnectivity and hypoconnectivity patterns (17–19). These abnormalities are mainly concentrated in the social brain network (e.g., amygdala, superior temporal sulcus), default mode network(e.g., Medial Prefrontal Cortex, Posterior Cingulate Cortex), and sensorimotor areas (e.g., precentral gyrus, paracentral lobule). However, the neurobiological mechanisms underlying SST-induced improvements in social deficits among individuals with ASD remain insufficiently characterized. To date, only one neuroelectrophysiological study has investigated the brain mechanisms by which social skills training improves social interaction difficulties in individuals with ASD (20). The study demonstrated that SST induced measurable neuroplastic changes, particularly enhanced left-lateralized frontal alpha asymmetry on electroencephalography, which correlated with improved social responsiveness and reduced social avoidance behaviors in adolescents with ASD.

Although previous studies have identified abnormalities in local brain function in ASD, it remains unclear whether SST can induce ReHo changes and whether these changes are associated with improvements in social deficits. Therefore, this study aimed to explore the changes of local brain function in whole brain using R-fMRI technology with a particular focus on ReHo in adolescents and adults with ASD after SST, and explore the relationship between changes of local brain function and improvements in social deficits, thereby improving understanding of neural mechanisms of SST in adolescents and adults with ASD.

## 2 Materials and methods

### 2.1 Participants

This study encompassed 22 participants in the training group and 22 participants in the control group. Participants were individuals with ASD who attended Peking University Sixth Hospital from September 2017 to March 2019, adhering to the following inclusion criteria: 1. Diagnosed as ASD according to DSM-5 (1) by two experienced child and adolescent psychiatrists; 2. Aged between 12 and 30; 3. Total IQ score ≥ 70, as measured by the Chinese-Wechsler Intelligence Scale for Children (C-WISC) (21) or the Wechsler Adult Intelligence Scale-Revised in China (WAIS-RC) (22); 4. Han ethnicity; 5. Right-handed; 6. Capable of remaining still for 30 minutes and cooperating with MRI examination; 7. Able to maintain and complete the full duration of social skills training; 8. Able to remain supine quietly for 30 minutes and cooperate with MRI examinations; 9. Consent from both the subjects and their statutory guardians to participate. Exclusion criteria included: 1. A clinical diagnosis or assessment using the Schedule for Affective Disorders and Schizophrenia for School-Age Children-Present and Lifetime Version (K-SADS-PL) (23) indicating other severe mental disorders such as schizophrenia, bipolar disorder, etc.; 2. Severe and unstable physical illnesses, neurological disorders, or brain injuries such as epilepsy, encephalitis, myocarditis, etc.; 3. History of alcohol or substance abuse or dependence; 4. Presence of metal objects in the body such as pacemakers, insulin pumps, artificial heart valves, etc.; 5.Participants whose original treatment (such as pharmacological treatments and/or general supportive counseling not focused on social skills) was unstable during the training period or who received new additional treatments; 6.Those who had received physical therapies (such as repetitive transcranial magnetic stimulation treatment and electroencephalogram treatment) or participated in any other structured social communication therapies within the two months prior to the SST. The inclusion and exclusion criteria for the control group were identical to those for the training group, except those participants did not receive social skills training. Participants were assigned to the training or waitlist (control) group sequentially by enrollment order. This procedure was applied separately to adolescent (12–17 years) and adult (over 18 years) cohorts.

After quality control of the MRI data (refer to fMRI data preprocessing), 20 persisted in the training group and 18 in the control group. No statistically significant differences were recorded between the two groups concerning gender [(16:4) vs. (15:3); χ2 = 1.05, P = 0.591], age [(16.82 ± 5.11) vs. (17.20 ± 4.09); t = -0.32, P = 0.754], full-scale IQ [(93.35 ± 16.78) vs. (101.06 ± 19.32); t = -1.56, P = 0.127], verbal IQ [(96.40 ± 18.76) vs. (103.56 ± 22.19); t = -1.28, P = 0.209], and performance IQ [(90.15 ± 18.59) vs. (97.11 ± 17.16); t = -1.39, P = 0.174] (refer to **Table 1**). Among them, 2 in the training group and 2 in the control group were on psychiatric medications for conditions including anxiety, depression, and emotional instability (refer to **supplementary Table 1**). The types and doses of medication remained stable throughout the study duration. No participant was receiving general supportive counseling.

**Table 1:** Comparison of general data between the training and control groups.

Demographic characteristics	Training group (n = 20)	Control group (n = 18)	t/χ^2^/z	P
Gender (male: female)	16:4	15:3	1.052	0.591
Age (years)	16.82 ± 5.11	17.20 ± 4.09	-0.316	0.754
FIQ	93.35 ± 16.78	101.06 ± 19.32	-1.564	0.127
VIQ	96.40 ± 18.76	103.56 ± 22.19	-1.279	0.209
PIQ	90.15 ± 18.59	97.11 ± 17.16	-1.388	0.174
FD	0.089 ^a^	0.059^a^	0.512	0.596^b^

FIQ, Full Intelligence Quotient; VIQ, Verbal Intelligence Quotient; PIQ, performance Intelligence Quotient; FD, framewise displacement.

^a^ median; ^b^ using nonparametric statistical test.

The study was approved by the Ethics Committee of Peking University Sixth Hospital. Participants were informed that the study aimed to investigate the change of brain function and behavior in ASD during social skills training. Control group would be offered the opportunity to receive the SST intervention after completing the study period as an ethical consideration. Participants and their guardians received full disclosure regarding the study and its objectives, ensuring voluntary participation. Both the participants and their guardians signed the written informed consent forms.

### 2.2 Social skills training program and implementation

Based on China’s cultural context and prior clinical and training experiences, our research team developed a culturally adapted ‘Social Skills Training Manual’, referring to the evidence-based PEERS^®^ intervention (24), originally developed at the University of California, Los Angeles (UCLA). The manual we developed is specifically designed for adolescents and adults with ASD, which has been validated to exhibit significant improvement in social interaction impairments among children, adolescents and adults with autism (14, 15).

​​The SST program targeted social skill deficits in individuals with ASD, utilizing games, role-playing, and interactive discussions to enhance social interaction abilities. The intervention was delivered via group therapy sessions that included individuals with ASD and their caregivers. During sessions, participants with ASD and their parents were assigned to separate therapeutic groups. Each participant group comprised 6–10 individuals with ASD (participants aged ≥18 years were grouped separately), and parent groups consisted of primary caregivers. Participant groups were led by 2–3 licensed child psychiatrists and autism rehabilitation therapists, who simultaneously conducted parent training sessions. ​​For the patient group, the program focused on common social difficulties and the core social skills needed to build and maintain friendships, such as communication strategies, peer selection, conflict resolution (e.g., addressing rumors), non-verbal communication, and boosting self-confidence and social motivation.​ Parent training involved reviewing weekly session content, discussing homework completion, assigning new homework, and providing strategies to support participants and reinforce skills in home settings. Facilitators tracked homework compliance through electronic communication (e.g., email, WeChat(微信)) or phone calls and addressed implementation challenges.

The training group participated in 14 weeks of social skills training conducted weekly, with each session lasting approximately 90 minutes, while the control group received no intervention. Prior to enrollment, both groups were maintaining their existing interventions, which primarily consisted of stable pharmacological treatments. These interventions remained unchanged throughout the study period, and no participants received other structured social skills interventions during this time. Neuroimaging data and behavioral assessments, including the Social Responsiveness Scale (SRS) and Aberrant Behavior Checklist (ABC), were obtained from both groups at baseline (pre-intervention) and endpoint (post-intervention) (See **Supplementary Material** for detailed description of the SST intervention).

### 2.3 Magnetic resonance imaging data acquisition

#### 2.3.1 Data acquisition process and scanning parameters

All imaging data were acquired on the GE Discovery 3T MR750 scanner at the Imaging Center of Peking University Sixth Hospital. During the scan, subjects were instructed to remain quiet, keep their eyes closed, stay awake, try to keep their head still, and avoid engaging in systematic thinking. A high-resolution T1-weighted structural image was obtained through a gradient-echo sequence (176 slices, layer thickness = 1.0mm, repetition time (TR) = 6.7ms, echo time (TE) = 3.1ms, flip angle = 8 degrees, field of view (FOV) = 256×256mm, inversion time (TI) = 450ms). An 8-min rest scan comprising 240 echo-planar imaging (EPI) functional volumes was also collected (43 slices, layer thickness = 4mm, TR = 2000ms, TE = 30ms, FOV = 220×220mm, matrix = 64×64, flip angle = 90 degrees, voxel size = 3.4×3.4×3.2mm^3^).

#### 2.3.2 fMRI data preprocessing

The fMRI data underwent preprocessing using the Data Processing Assistant for Resting-State fMRI (DPARSF, http://rfmri.org/DPARSF) tool (25), predicated on the Data Processing & Analysis for Brain Imaging (DPABI, http://rfmri.org/DPABI) toolbox (26) and Statistical Parametric Mapping 8 (SPM8, http://www.fil.ion.ucl.ac.uk/spm). The first 10 time points of the fMRI data were removed, and temporal slice timing correction was subsequently executed on the remaining 230 time points. Head motion was corrected by a six-parameter (rigid body) linear transformation, employing a two-pass procedure (25). After realignment of the structural and functional images and unified segmentation (27) on the T1 image, spatial normalization was performed by transforming the functional data from individual space to the Montreal Neurological Institute (MNI) standard space using the DARTEL tool (28). The Friston 24-parameter model was used to remove motion effects (29). Additionally, the white matter and cerebrospinal fluid signals were regressed out from each voxel’s time course, with a linear trend employed as a regressor to remove low-frequency drift in the blood oxygen level-dependent (BOLD) signal (without global signal regression (GSR)). Finally, all images were filtered by temporal bandpass filtering (0.01–0.1 Hz) to reduce the effect of low-frequency drift and high-frequency physiological noise. To account for the impact of head motion on fMRI data, the Jenkinson average head motion parameter (framewise displacement, FD) was adopted as a metric of head motion to screen participants. No additional scrubbing (e.g., spike regression) was performed. Participants with a mean FD value greater than 0.292 (the mean FD value of all participants plus two standard deviations, i.e., 0.074 + 2 * 0.109) were consequently excluded. A total of 6 participants (2 from the training group, 4 from the control group) were excluded due to excessive head motion.

#### 2.3.3 The calculation of the regional homogeneity

Our study employed the Regional Homogeneity (ReHo) computation approach (16) to measure the local brain functional activity in individuals with ASD, before and after SST. ​​ReHo was selected over alternative metrics like amplitude of low-frequency fluctuations (ALFF) or fractional ALFF (fALFF) due to its sensitivity to localized neural synchronization within functionally homogeneous regions (16). While ALFF/fALFF quantify regional spontaneous neural activity intensity (30), ReHo captures the temporal coherence of BOLD signals among neighboring voxels, making it ideal for detecting SST-induced changes in local functional integration. Prior ASD studies further validate ReHo’s utility in identifying atypical connectivity patterns linked to social deficits (17, 18).​​ReHo was executed by computing the Kendall’s coefficient of concordance (KCC) of the fMRI time series for a specific voxel in relation to its neighboring 26 voxels. Subsequently, the KCC value of each voxel was divided by the mean KCC of the entire brain to derive a standardized ReHo map, thereby mitigating the influence of individual variability. Finally, a 4mm FWHM smoothing kernel was utilized to perform spatial smoothing.

### 2.4 Tools

#### 2.4.1 Wechsler intelligence scale for children and Wechsler adult intelligence scale

The Wechsler Intelligence Scale for Children (C-WISC) (21) assesses the intelligence of children aged 6 to 16, while the Wechsler Adult Intelligence Scale-Revised (WAIS-RC) (22) evaluates intelligence in adolescents and adults aged 17 and above. Scaled scores are calculated for each subtest as per the manual, resulting in the Verbal Intelligence Quotient (VIQ), Performance Intelligence Quotient (PIQ), and Full Intelligence Quotient (FIQ). A total IQ score of 85 or above is considered indicative of normal intelligence, 70–85 signifies borderline intelligence, while a score below 70 indicates an intellectual disability.

#### 2.4.2 Social responsiveness scale

The Social Responsiveness Scale comprises 65 items that evaluate ASD symptoms manifesting in natural settings. It is utilized to assess social skills in individuals with ASD and can also evaluate social interaction abilities in these individuals before and after treatment to determine the effectiveness of interventions. Higher scores correlate with more severe symptoms. The items are categorized into five subscales: social awareness, social cognition, social communication, social motivation, and autistic mannerisms. Parents, caregivers, or teachers familiar with the child’s social interactions over the past month in natural settings complete the scale. The scale has been translated into Chinese and validated, demonstrating strong reliability and validity (31). Additionally, prior research has employed the SRS to evaluate the effectiveness of interventions for adults with ASD (32).

#### 2.4.3 Aberrant behavior checklist

The Aberrant Behavior Checklist comprises 58 items across five subscales: irritability/aggression, lethargy/social withdrawal, stereotypic behavior, hyperactivity, and inappropriate speech. It requires parents, caregivers, or teachers to evaluate the patient’s behavior over the past month, serving as a tool to assess ASD symptoms in individuals aged three and above. This tool is widely used to evaluate intervention outcomes in ASD. The Chinese version of the checklist, introduced by Ma (33), has exhibited strong reliability and validity.

### 2.5 Statistical analysis

The primary behavioral outcomes were pre-specified as the scores of the ABC and SRS. For the pre-specified primary behavioral outcomes (the total and subscales scores of ABC and SRS), Linear Mixed Models (LMM) were employed. The model ​​included​​ group (training vs. control), condition (baseline vs. endpoint), group × condition interaction, ​​with​​ age, gender, and FIQ as covariates. Follow-up comparisons (paired and independent samples t-tests) and reduction rate analyses were conducted to detail specific patterns of change. The primary neuroimaging analysis was a whole-brain examination of ReHo. Statistical analysis was conducted on general data and imaging data using IBM SPSS 20.0 (Statistical Package for the Social Sciences) software package and DPABI software. P<0.05 was considered as statistically significant. (See **Supplementary Material** for detailed statistical analysis on general information and social skills training data).

First, a linear mixed model was conducted to explore the group × condition interaction effect of whole-brain ReHo values in both the training and control groups. The model ​​included​​ group (training vs. control), condition (baseline vs. endpoint), group × condition interaction, and average FD as fixed-effect factors, ​​with​​ age, gender, and FIQ as covariates. And Permutation Testing with Threshold-Free Cluster Enhancement (PT with TFCE) correction was applied for multiple comparisons. (See **Supplementary Material** for detailed statistical analysis on linear mixed model).

Subsequently, clusters with significant group × condition interaction were defined as masks and the average ReHo values for each subject within each mask were extracted. Paired samples t-tests were conducted to compare ReHo values pre- and post-treatment in the training group, as well as at baseline and endpoint in the control group.

Finally, linear regression, with age, gender, FIQ, and average FD as covariates, was utilized to explore the relationship between changes in ReHo and SRS and ABC scores before and after training. Specifically,​​ the changes in ReHo were set as predictors, and the change scores in the ABC and SRS scales were set as outcome variables. To account for multiple comparisons across the five clusters for each behavioral scale, a Bonferroni correction was applied, setting the significance threshold at α = 0.01 (0.05/5) for each set of five tests.

A *post-hoc* sensitivity analysis was performed by re-running our primary statistical models after excluding the four participants (two from the training group and two from the control group) who were on stable psychotropic medication during the study period. This analysis involved re-running the key statistical models (the paired t-tests for the primary behavioral outcomes/the linear mixed model for ReHo changes and the linear regression between alterations in ReHo and changes in symptom severity pre- and post-training within the training group) after excluding the four participants.

## 3 Results

### 3.1 The group × condition interaction of ReHo

**Figure 1:**
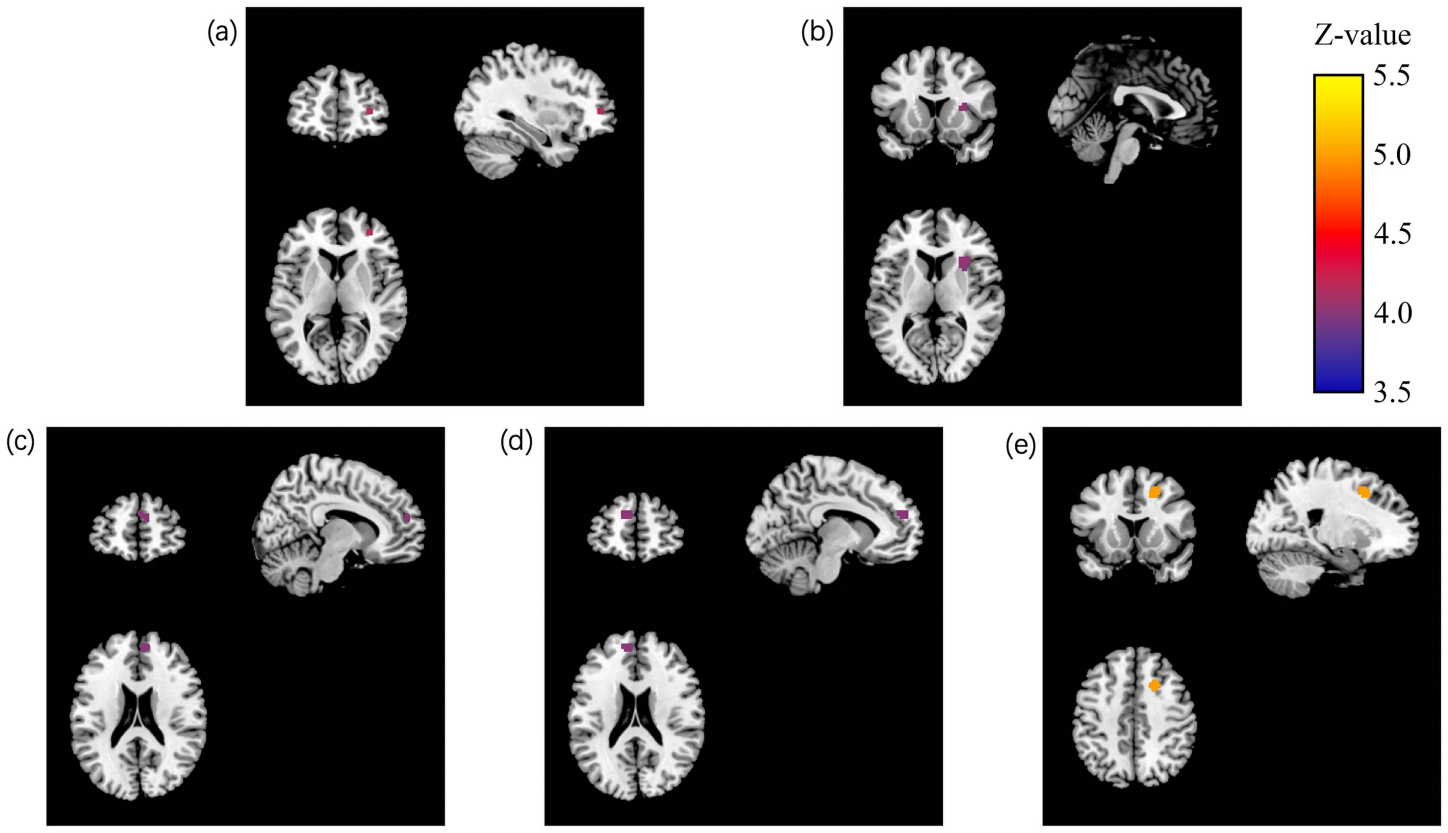
Brain regions of the training and control groups showing significant group × condition interaction effects. Images of brain regions exhibiting a significant group x condition interaction effect (PT with TFCE correction, p < 0.05) were displayed using MRIcroN software. The coronal view appears on the top left, the sagittal view on the top right, and the axial view on the bottom left. Cluster 1 (right medial frontal gyrus) is denoted by **(a)**, cluster 2 (right insula) by **(b)**, cluster 3 (right medial superior frontal gyrus) by **(c)**, cluster 4 (left medial superior frontal gyrus) by **(d)**, and cluster 5 (right medial frontal gyrus) by **(e)**. Color bar indicating Z-values ranging from 3.5 to 5.5, representing the statistical significance of the group × condition interaction effects (PT with TFCE correction, p < 0.05).

The linear mixed model analysis revealed significant group × condition interaction effects on the ReHo values within both the training and control groups. Specifically, five clusters demonstrated significant group × condition interaction effects (PT with TFCE correction, p < 0.05; see **Table 2**, **Figure 1**).

**Table 2:** Brain regions of the training and control groups showing significant group × condition interaction effects.

Clusters	Cluster size (Voxels)	MNI coordinate of peak point				Peak intensity (z)	Cluster regions (AAL)
		X	Y	Z	Brain area(AAL)		
Cluster1	1	33	48	6	Right medial frontal gyrus	4.2577	Right medial frontal gyrus
Cluster2	14	0	15	9	Right Sub-lobar Extra-Nuclear	4.2297	Right Extra-Nuclear, right insula, right putamen
Cluster3	6	9	54	21	Right medial superior frontal gyrus	4.0033	Right medial superior frontal gyrus
Cluster4	16	-9	54	21	Left medial superior frontal gyrus	3.964	Left medial superior frontal gyrus, left dorsolateral superior frontal gyrus
Cluster5	12	21	15	45	Right medial frontal gyrus	5.022	Right medial frontal gyrus, right medial superior frontal gyrus

MNI, Montreal Neurological Institute; X, Y, Z, peak coordinate of cluster (based on MNI coordinate system); AAL, Anatomical Automatic Labeling.

### 3.2 Comparison of ReHo between the training and control groups at baseline and endpoint

An independent sample t-test was conducted to compare the ReHo value of each cluster at baseline and endpoint between the training group and the control group. At baseline, the results indicated that, except for cluster 2 (t (36) = 2.914, p = 0.006, Hedges’ g= 0.955) and cluster 3 (t (36) = 3.242, p = 0.003, Hedges’ g= 1.070), there were no significant differences in ReHo values between the two groups for the remaining clusters (t (36) = -0.849 to 1.767, all p>0.05) (see **Table 3**).

**Table 3:** ReHo values at baseline and endpoint, and results of within-group and between-group comparisons.

A. Descriptive data: mean scores at baseline and endpoint.					
ReHo values	Training group (n = 20)		Control group (n = 18)		
	Baseline	Endpoint	Baseline	Endpoint	
Cluster 1	1.11 ± 0.43	0.76 ± 0.38	1.24 ± 0.46	1.30 ± 0.50	
Cluster 2	-0.22 ± 0.44	-0.52 ± 0.24	-0.58 ± 0.30	-0.37 ± 0.27	
Cluster 3	1.11 ± 0.34	0.79 ± 0.31	0.73 ± 0.37	0.96 ± 0.50	
Cluster 4	1.25 ± 0.34	0.92 ± 0.39	1.08 ± 0.33	1.19 ± 0.48	
Cluster 5	0.53 ± 0.41	0.22 ± 0.45	0.30 ± 0.39	0.47 ± 0.47	
B. Statistical comparisons (effect sizes with 95% confidence intervals).					
Comparison type	Cluster 1	Cluster 2	Cluster 3	Cluster 4	Cluster 5
Within-training (Paired t)					
*t* (19)	4.833	2.777	3.932	4.700	3.774
*p*	<0.001**	0.012*	0.001**	<0.001**	0.001**
Cohen’ s d [95% CI]	0.862 [0.214, 1.51]	0.847 [0.2, 1.494]	0.985 [0.329, 1.641]	0.902 [0.251, 1.553]	0.721 [0.081, 1.361]
Within-control (Paired t)					
*t* (17)	-0.653	-2.346	-2.568	-1.489	-2.657
*p*-value	0.523	0.031*	0.020*	0.155	0.017*
Cohen’ s d [95% CI]	-0.125 [-0.779, 0.529]	-0.737 [-1.412, -0.062]	-0.523 [-1.187, 0.141]	-0.267 [-0.923, 0.389]	-0.394 [-1.054, 0.266]
Between-group at baseline (indep. t)					
*t* (36)	-0.849	2.914	3.242	1.588	1.767
*p*	0.401	0.006**	0.003**	0.121	0.086
Hedges’ g [95% CI]	-0.306 [-0.946, 0.334]	0.955 [0.283, 1.627]	1.070 [0.389, 1.751]	0.507 [-0.14, 1.154]	0.580 [-0.082, 1.243]
Between-group at endpoint (indep. t)					
*t* (36)	-3.790	-1.768	-1.282	-1.885	-1.676
*p*	0.001**	0.086	0.208	0.068	0.102
Hedges’ g [95% CI]	-1.236 [-1.953, -0.519]	-0.577 [-1.234, 0.081]	-0.418 [-1.077, 0.241]	-0.616 [-1.284, 0.052]	-0.548 [-1.210, 0.113]

Data are presented as mean ± SD. For within-group comparisons (paired t-tests), effect sizes are reported as Cohen’s d with 95% confidence intervals (CI). For between-group comparisons (independent t-tests), effect sizes are reported as Hedges’ g with 95% CI. * p<0.05;**p<0.01.

At the endpoint, the training group exhibited significantly reduced ReHo values in cluster 1 compared to the control group (t (36) = -3.790, p = 0.001, Hedges’ g= -1.236), which indicates a statistically significant difference. No significant differences were observed for the remaining clusters (t (36) = -1.864 to -1.251, all p > 0.05; see **Table 3**).

### 3.3 Within-group comparison of ReHo in the training and control groups at baseline and endpoint

Paired t-tests were conducted on the ReHo values of each cluster within the training group at both baseline and endpoint. The results revealed that following 14 weeks of training, the ReHo values of each cluster decreased significantly compared to baseline. (t (19) = 2.777 to 4.833, all p<0.05, Cohen’ s d=0.721 to 0.985) (see **Table 3**).

We also performed paired t-tests on the ReHo value of each cluster within the control group at baseline and endpoint. The findings indicated that at endpoint, Clusters 2 (t (17) = -2.346, p = 0.031, Cohen’ s d=-0.737), 3 (t (17) = -2.568, p = 0.020, Cohen’ s d= -0.523), and 5 (t (17) = -2.657, p = 0.017, Cohen’ s d= -0.394) exhibited a significant increase compared to baseline. However, no significant differences in the ReHo values of Clusters 1 and 4 were observed between baseline and endpoint (t = -1.498 to -0.653, all p>0.05) (see **Table 3**).

### 3.4 Behavioral outcomes

#### 3.4.1 Linear mixed model analysis of ABC and SRS scale scores

The behavioral outcomes were analyzed using a Linear Mixed Model (LMM) to evaluate the effects of Time (Baseline, Endpoint), Group (Training, Control), and their interaction. A significant main effect of group was found for the ABC total score (F (1, 34.84) = -2.198, p = 0.035, Partial η²= 0.059), as well as for the Irritability/Aggression (F (1, 35.08) = -2.978, p = 0.005, Partial η²=0.078) and Hyperactivity (F (1, 34.41) = -2.443, p = 0.020, Partial η²=0.066) subscales. However, other main effect of group or the critical Time × Group interaction effect was not statistically significant for any of the ABC or SRS total or subscales scores in this model (all p > 0.05).

#### 3.4.2 Comparison of ABC and SRS scale scores between the training and control groups at baseline and endpoint

Independent sample t-tests were administered to compare the baseline and endpoint scores of the ABC and SRS scales, for both total and factor scores, between the training and control groups. At baseline, the results revealed no significant differences in the total and factor scores of the ABC and SRS scales between the groups (t (36) = -1.636 to 0.723, all p > 0.05; see **Supplementary Table 2**).

At the endpoint, the results showed that the training group had significantly lower scores in the irritability (t (36) = 5.615, p = 0.005, Hedges’ g= -1.104), hyperactivity factor (t (36) = 5.270, p = 0.026, Hedges’ g= -0.819), and total score of the ABC scale (t (36) = 4.689, p = 0.026, Hedges’ g= -0.805) compared to the control group, indicating statistically significant differences. No statistically significant differences were observed for the remaining factors of the ABC scale or any factors and the total score of the SRS scale (t (36) = 0.006 to 3.277, all p > 0.05; see **Supplementary Table 2**).

#### 3.4.3 Within-group comparison of ABC and SRS scores within the training and control groups at baseline and endpoint and the intergroup differences

A paired t-test was employed to compare the total and factor scores of the ABC scale between the training group’s baseline and endpoint assessments. The analysis demonstrated that following 14 weeks of training, the total score of the ABC scale (t (19) = 2.329, p = 0.033, Cohen’s d= 0.501) and the social withdrawal factor score (t (19) = 2.806, p = 0.013, Cohen’s d= 0.626) were significantly reduced compared to baseline. However, no significant differences were found in the scores of the irritability, stereotypic behavior, hyperactivity, and inappropriate speech factors between baseline and endpoint assessments (t (19) = 1.000 to 2.111, all p > 0.05; see **Supplementary Table 2**).

Paired t-tests were similarly conducted on the total and factor scores of the ABC scale at baseline and endpoint in the control group. The findings indicated that at the endpoint, the stereotypic behavior factor score of the ABC scale was significantly lower than at baseline (t (17) = 2.426, p = 0.027, Cohen’s d= 0.253). Nevertheless, no significant differences were observed in the total score and the scores of the irritability, social withdrawal, hyperactivity, and inappropriate speech factors between baseline and endpoint assessments (t (17) = 0.421 to 1.203, all p > 0.05; see **Supplementary Table 2**).

Additionally, independent sample t-tests were applied to compare the reduction rates from baseline to endpoint for each factor and the total score of the ABC scale between the training and control groups. Results showed a significantly higher reduction rate in the hyperactivity factor score for the training group compared to the control group [training group vs. control group: (0.19 ± 0.52) vs. (-0.71 ± 1.66); t (36) = 2.127, p = 0.047, Hedges’ g= 0.732 [0.074, 1.39]]. No significant differences were found in the reduction rates for the other factors or the total ABC score between the two groups (p > 0.05).

A paired t-test was conducted to compare the total and factor scores of the SRS scale between the training group’s baseline and endpoint assessments. The findings revealed statistically significant reductions after 14 weeks of training in the total SRS score (t (19) = 2.756, p = 0.013, Cohen’s d=0.690) and the scores for social awareness (t (19) = 3.577, p = 0.002, Cohen’s d=0.703), social cognition (t (19) = 2.950, p = 0.008, Cohen’s d=0.618), social communication (t (19) = 2.933, p = 0.009, Cohen’s d=0.716), and social motivation (t (19) = 2.333, p = 0.031, Cohen’s d=0.395), all of which were lower than baseline. However, the autistic mannerisms factor showed no statistically significant difference between baseline and endpoint (t (19) = 0.559, p > 0.05; see **Supplementary Table 2**).

Similarly, a paired t-test was executed on the total and factor scores of the SRS scale for the control group at baseline and endpoint. The results indicated statistically significant reductions in the scores for social communication (t (17) = 2.631, p = 0.018, Cohen’s d=0.416) and social motivation (t (17) = 2.135, p = 0.048, Cohen’s d=0.269) at the endpoint, both lower than baseline.

We also conducted independent sample t-tests to analyze the reduction rates from baseline to endpoint for each factor and the total score of the SRS scale between the training and control groups. The results revealed that the reduction rate of the social awareness factor in the training group was significantly higher than that in the control group [training group vs. control group: (0.17 ± 0.22) vs. (-0.04 ± 0.18); t (36) = 3.196, p = 0.003, Hedges’ g= 1.045 [0.366, 1.724]]. No significant differences were observed in the reduction rates for the remaining factors or the total SRS score between the two groups (p > 0.05).

### 3.5 The correlation between changes in ReHo and changes in symptom severity within the training group pre- and post-training

Linear regression was employed to explore the correlation between alterations in ReHo and changes in symptom severity pre- and post-training within the training group. The changes in ABC and SRS scores pre- and post-training were utilized as dependent variables, whereas age, gender, total IQ, and head movement parameters served as covariates. Furthermore, the difference in baseline-to-endpoint ReHo values was incorporated as the independent variable within the linear regression model. Based on our primary behavioral results (Section 3.4), the dependent variables are limited to the behavioral outcomes that showed significant improvement post-SST in training group: the ABC total and social withdrawal scores, and the SRS total, social cognition, social communication, and social motivation scores. After applying Bonferroni correction for multiple comparisons across the five clusters (significance threshold set at p < 0.01), a significant linear relationship emerged between the changes in total ABC scale scores pre- and post-training and the difference in ReHo values of Cluster 1 (F (9, 37) = 3.128, P = 0.010, adjusted R² = 0.350), yielding a partial regression coefficient of 31.664 (see **Table 4**). However, no significant linear relationships were observed between the variations in other scores and the baseline-to-endpoint ReHo value differential (F (9, 37) = -2.032 to 2.019, all p>0.01).

**Table 4:** The linear regression between changes in ReHo and changes in symptom severity within the training group pre- and post-training.

Scales	The baseline-endpoint differences of scales	The baseline-endpoint differences of ReHo	Partial regression coefficient	Normalized partial regression coefficient	t	P
ABC	Social withdrawal	Cluster1	4.952	5.107	0.970	0.353
		Cluster2	2.128	0.221	0.578	0.575
		Cluster3	0.115	0.010	0.036	0.972
		Cluster4	-1.880	-0.128	-0.469	0.648
		Cluster5	-0.687	-0.052	-0.152	0.882
	The total score of ABC	Cluster1	31.664	0.610	3.128	0.010*
		Cluster2	-1.373	-0.037	-0.120	0.907
		Cluster3	-7.123	-0.183	-0.890	0.392
		Cluster4	-11.460	-0.237	-1.146	0.276
		Cluster5	0.376	0.009	0.032	0.975
SRS	Social awareness	Cluster1	-1.828	-0.288	-1.099	0.290
		Cluster2	-1.915	-0.445	-1.397	0.184
		Cluster3	0.343	0.059	0.249	0.807
		Cluster4	-0.441	-0.066	-0.277	0.786
		Cluster5	-2.245	-0.402	-1.630	0.125
	Social cognition	Cluster1	5.396	0.445	1.683	0.114
		Cluster2	1.398	0.170	0.475	0.642
		Cluster3	-3.166	-0.286	-1.185	0.256
		Cluster4	1.360	0.107	0.424	0.678
		Cluster5	-3.038	-0.285	-1.036	0.318
	Social communication	Cluster1	9.608	0.354	2.009	0.064
		Cluster2	-0.190	-0.010	-0.042	0.976
		Cluster3	3.334	0.134	0.788	0.444
		Cluster4	0.618	0.022	0.124	0.903
		Cluster5	-6.906	-0.289	-1.597	0.133
	Social motivation	Cluster1	3.013	0.248	0.878	0.365
		Cluster2	1.914	0.233	0.653	0.524
		Cluster3	3.837	0.346	1.467	0.164
		Cluster4	-3.243	-0.255	-1.040	0.316
		Cluster5	-5.417	-0.508	-2.014	0.064
	The total score of SRS	Cluster1	26.278	0.405	2.019	0.063
		Cluster2	4.790	0.109	0.386	0.705
		Cluster3	6.299	0.106	0.540	0.598
		Cluster4	-2.495	-0.037	-0.184	0.857
		Cluster5	-22.883	-0.401	-2.032	0.062

* indicates significance surviving Bonferroni correction for five tests per behavioral scale (p < 0.01).

A sensitivity analysis excluding participants on psychotropic medication confirmed that the primary neuroimaging and behavioral findings persisted substantially unchanged except for the linear relationship between the changes in total ABC scale scores pre- and post-training and the difference in ReHo values of Cluster 1 (see **Supplementary Material**).

## 4 Discussion

Social interaction deficits constitute one of the core features of ASD. It has been proved that social interaction deficits are associated with dysfunctions in local brain function. SST is the principal intervention method targeting social interaction deficits. Our study demonstrated that SST may modulate local functional connectivity (measured by R-fMRI) within the right medial frontal gyrus, right insula, right superior frontal gyrus, and left superior frontal gyrus, which are components of the social brain network. Significant group × condition interaction effects was found on ReHo within these clusters between the training and control groups. Our study also suggested that social skills training significantly enhances social perception, social cognition, overall social functioning, reduces social withdrawal, decreases hyperactivity, and mitigates overall symptoms in adolescents and adults with ASD. Further analysis revealed an exploratory linear relationship between the change in overall symptoms of ASD and the alteration in ReHo values of cluster 1 (right medial frontal gyrus).

​​To our knowledge, no other studies have investigated the changes in brain function following SST in ASD, except for Van Hecke et al. (20), who utilized electroencephalography to examine the neuroplasticity during SST. Our study employed R-fMRI to investigate alterations in local brain function following SST aimed at enhancing social interaction abilities in adolescents and adults with ASD. The results demonstrated significant differences in ReHo values between the two groups across five clusters located in the left and right superior frontal gyri, right medial frontal gyrus, and right insula regions. These differences, observed at the endpoint, were attributed to group × condition interaction effects. At baseline, no significant differences in ReHo values for cluster 1 (right medial frontal gyrus) were observed between the groups; however, the ReHo value for cluster 1 in the training group was significantly lower than that in the control group at endpoint. Notably, Cluster 1 was spatially limited (a single voxel). Although the spatial extent of this cluster warrants a cautious interpretation, its location within a critical social brain region lends biological rationality to the finding and highlights a precise area for future investigations. Further analysis revealed that the ReHo values of the five clusters were significantly reduced in the training group at the endpoint relative to pre-training levels. In contrast, the control group exhibited significantly increased ReHo values in cluster 2 (right insula), cluster 3 (right medial superior frontal gyrus), and cluster 5 (right medial frontal gyrus) compared to baseline.

Our study’s findings demonstrated significant differences between the training and control groups in clusters located in the superior frontal gyrus (SFG), medial frontal gyrus (MFG), and insula. Functionally, these regions subserve distinct neurocognitive processes such as face recognition (34), theory of mind (35), and executive function (36). ​​The SFG​​ supports executive control and social decision-making by integrating contextual cues and suppressing irrelevant stimuli (36); the MFG mediates theory of mind and conflict monitoring (35); while the insula integrates interoceptive signals for emotional awareness (37). Previous neuroimaging studies have consistently demonstrated abnormal functional connectivity within the SFG, MFG, and insula in ASD (37–39).​Previous research has also demonstrated that individuals with ASD exhibit significant abnormalities in ReHo within the SFG, MFG, and insula compared to typically developing (TD) (18, 19). Furthermore, Individuals with ASD displayed lower ReHo values in sensory processing areas and elevated ReHo values in regions involved in complex information processing compared to TD (17). These findings suggest that SST employed in our study may improve the identified regional functional abnormalities within the aforementioned brain regions in adolescents and adults with ASD. Furthermore, Dajani and Uddin (17) identified significant atypical developmental patterns of ReHo in ASD. While TD individuals typically show decreasing ReHo values from childhood to adulthood, individuals with ASD often exhibit an opposite pattern, with ReHo values increasing from childhood into adolescence and adulthood. Although conducted over a brief period, our study revealed an increasing trend in ReHo values within the three brain regions of the control group, consistent with the atypical developmental pattern observed in prior ASD research. The declining trend in ReHo values of the training group——such as the left and right SFG, right MFG, and right insula——confirmed that the social skills training employed in this study could modify the local functional connectivity in ASD, may reverse the atypical developmental pattern, and improve social interaction difficulties and other symptoms. Previous studies have found that the SFG, MFG, and insula are recognized as components of the ‘social brain’ network (40), which encompasses interconnected structures (e.g., amygdala, hippocampus, ventromedial prefrontal cortex, anterior and posterior temporal sulcus, angular gyrus) critical for mediating social cognition. The observed SST-induced declining trend in ReHo values within these specific regions points towards a normalization of local functional organization in crucial nodes of the social brain network, offering a potential neurobiological mechanism for the social interaction impairments.

Regarding symptom evaluation, this study found that SST can effectively improve overall symptoms and social interaction deficits in adolescents and adults with ASD. First, linear mixed model analysis was used to evaluate symptom, which revealed significant main effects of group for the ABC total score as well as for the Irritability/Aggression and Hyperactivity subscales, suggesting that the training group maintained consistently lower symptom levels throughout the study period when compared to the control group. However, the critical group × condition interaction effect—which directly tests whether the rate of change over time differs between groups—was not statistically significant for either scale. The absence of interaction effect may be attributed to the modest sample size, which can limit the statistical power of LMM to detect interaction effects, known to be more challenging to detect than main effects. Furthermore, follow-up t-tests indicated that both groups showed statistically significant improvements over time, although the training group improved on more subscales than the control group. Improvements in the control group, potentially due to factors such as natural development, could have attenuated the model’s sensitivity to a specific intervention effect. To complement the LMM and directly test the efficacy of the intervention at pre- and post-treatment time points, we therefore employed our pre-specified planned comparisons (paired and independent t-tests). T-tests showed that the training group demonstrated significantly greater reductions in ABC total scores and irritability/hyperactivity subscales versus controls post-intervention. Within the training group, significant pre-post reductions occurred in ABC total/social withdrawal scores and SRS total/social awareness/cognition scores. The control group also demonstrated statistically significant improvements on the ABC stereotypic behavior, SRS social communication and social motivation factors. However, these limited changes may be attributable to non-specific factors, such as practice effects from repeated assessment, natural development over the course of the study, or the general supportive environment of participation. Crucially, unlike the training group, the control group did not exhibit concurrent, broad-based improvements across multiple behavioral domains, including the ABC total score, social withdrawal, SRS total score, social awareness, and social cognition. This pattern of results indicates that the comprehensive benefits observed in the training group are specifically linked to the SST intervention. The ABC is used to evaluate all core symptoms and common emotional and behavioral problems associated with ASD; the decrease in its total score and the factor scores for irritability, hyperactivity, and social withdrawal indicates that the SST method employed in this study can effectively alleviate overall symptoms in adolescent and adult ASD patients. This finding aligns with previous research (13, 15, 32). To comprehensively assess improvements in social interaction deficits among ASD patients, this study further utilized the SRS. The significant reductions in the training group’s total SRS scores, as well as in the social awareness and social cognition factor scores, indicate that the SST not only alleviates social interaction deficits but also promotes the development of social perception and social cognition, thus improving social skills from multiple dimensions. These results reinforce the positive role of SST in enhancing the social skills of individuals with ASD, a conclusion supported by extensive prior research (15, 20, 41, 42), while underscoring the need for future studies with larger samples to more powerfully test longitudinal interaction effects using models such as LMM.

Our study employed linear regression to investigate the correlation between symptom amelioration in ASD and alterations in ReHo. ​​Statistically significant reductions in ABC/SRS scores (pre-post) were designated as dependent variables, while the change in baseline-to-endpoint ReHo values served as independent variables, to quantify relationships between changes in ReHo and symptom improvement in ASD. A significant negative correlation was identified between the variance in total scores of the ABC scale pre- and post- training within the training group and the differential ReHo value of cluster 1 (right MFG). While cluster 1 was spatially limited to a single voxel, the observed correlation suggests an exploratory association between alterations in ReHo of the right MFG and changes in autism symptoms among ASD patients. The frontal medial gyrus constitutes a vital component of the social brain, with previous research demonstrating functional abnormalities in this region among individuals with ASD. For instance, Koshino et al. (43) discovered reduced activation levels in the frontal medial gyrus of ASD patients compared to TD. Several studies have likewise reported abnormal functional connectivity between the frontal medial gyrus and other brain regions linked to the social brain in ASD individuals. Fishman, Keown, Lincoln, Pineda, and Muller (44) reported enhanced functional connectivity between the posterior cingulate/precuneus and the right MFG in ASD adolescents relative to TD, whereas Libero et al. (45) identified diminished connectivity between the right superior temporal sulcus and the frontal medial gyrus in ASD patients in contrast to TD. Previous research has demonstrated close associations of the frontal medial gyrus with the face recognition (34), theory of mind (35), and executive function (36) capabilities, suggesting that functional abnormalities within this region may contribute to ASD symptoms by disrupting these cognitive processes. Therefore, enhancements in local brain function of the frontal medial gyrus through social skills training could potentially fortify executive function and theory of mind capabilities in ASD, indirectly ameliorating social interaction challenges and overall ASD symptoms in adolescents and adults. Additionally, Jiang et al. (18) revealed that ASD patients exhibited elevated ReHo values in the right MFG as opposed to TD, and the total score of the Autism Diagnostic Observation Schedule (ADOS) was positively correlated with ReHo values in this region, indicating that elevated ReHo values were associated with more severe autism symptoms. Dajani and Uddin (17) also demonstrated a positive correlation between ReHo values in ASD patients and the severity of ASD symptoms. These studies collectively imply that increased ReHo values within the social brain are positively correlated with the severity of ASD symptoms, thus providing additional support for our study’s findings that reductions in ReHo values are associated with symptomatic improvements in ASD.

### 4.1 Shortcomings of this study and future development direction

The study’s small sample size and exclusion of children under 12 years of age limit the generalizability of findings. Future investigations should strive to increase sample sizes and stratify participants by age to explore variations in brain function changes following SST among different age groups. Participants included individuals with comorbidities or those on psychiatric medications, which may influence brain function changes. Future work should categorize participants into those without comorbidities, those with comorbidities but not on medication, and those on medication to understand how these factors affect brain function. Intellectual disability is common in ASD, yet this study only included individuals with an IQ above 70. Future research should involve individuals with ASD who have intellectual impairments to investigate brain function changes in low-functioning ASD through SST. Given the higher prevalence of ASD in males compared to females, limited female participants were included. Future studies should aim to include more female participants to explore gender-specific brain function changes. Parent-reported outcomes (ABC, SRS) from unblinded caregivers introduces a potential for expectancy bias, which may have influenced the behavioral results. Future trials should mitigate this limitation by employing blinded independent evaluators or objective behavioral measures. The generalizability of our findings is limited due to the single-center design and the exclusive recruitment of right-handed individuals of Han ethnicity. Future multi-center studies with diverse, population-based samples that include individuals from various ethnicities and handedness groups are needed to validate and extend our conclusions. This study utilized ReHo as a sole indicator to examine brain function changes but did not explore other aspects such as brain functional connectivity. Future investigations should employ multimodal neuroimaging approaches such as seed-based connectivity to comprehensively map SST-induced brain function reorganization. The cluster in the right medial frontal gyrus (Cluster 1) was defined by a single voxel. While its location within a key node of the social brain network lends it biological plausibility, the small spatial extent may affect its reliability and warrants cautious interpretation and replication in future work. Lastly, no follow-up was conducted after training, leaving the sustainability of brain function changes uncertain. Future research should incorporate extended follow-up protocols to assess the long-term trajectory of brain function reorganization.

### 4.2 Conclusions

Overall, our study demonstrates that social skills training can alter local functional connectivity within the social brain of individuals with ASD, potentially reversing atypical developmental patterns in this population. After training, specific social brain regions demonstrated a decrease in ReHo values. Notably, the reduction in ReHo values in the right medial frontal gyrus may correlate with symptomatic improvement. Changes in ReHo within the social brain may be associated with the improvements in social interaction and overall symptoms observed following SST in individuals with ASD. Our study also affirms that social skills training constitutes an efficacious strategy to improve social interaction challenges and overall symptoms in adolescents and adults with ASD. These findings may provide insights into the neural mechanisms underlying SST-related changes of social deficits and symptom severity in ASD. Furthermore, our study offers valuable insights and evidence that may help advance the understanding of the neural pathophysiology of social interaction challenges in ASD and to inform the exploration of future treatment approaches for ASD.

## Data Availability

The original contributions presented in the study are included in the article/**Supplementary Material**. Further inquiries can be directed to the corresponding authors.
