# Supplementary material for: Changes in regional homogeneity of the social brain in individuals with autism spectrum disorder after social skills training
Source: Front Psychiatry. 2026 Mar 13;17:1674370. doi: 10.3389/fpsyt.2026.1674370 (PMC13021787; doi:10.3389/fpsyt.2026.1674370)
Supplement: Supplementary file 1 [file DataSheet1.pdf]

Changes in Regional Homogeneity of the social brain in individuals with Autism Spectrum Disorder after social skills training

Supplementary Material

1 Detailed Description of the SST Intervention

1.1 Intervention Content and Structure

The 14-week SST program was culturally and developmentally adapted from the evidence-based PEERS® curriculum. Each weekly 90-minute session followed a structured manual and focused on a specific core social skill module. The sequential modules were:

Session 1: Conversation Skills – Exchanging Information: Initiating conversations by finding common interests.

Session 2: Conversation Skills – Reciprocal Communication: Practicing turn-taking and maintaining balanced two-way dialogues.

Session 3: Conversation Skills – Electronic Communication: Appropriate use of texting and social media for social purposes.

Session 4: Identifying Peer Groups & Choosing Friends: Strategies for identifying shared interests and selecting potential friends from appropriate peer groups.

Session 5: Using Humor Appropriately: Differentiating between appropriate and inappropriate humor and understanding the context for joking.

Session 6: Initiating Conversations: Steps for starting a conversation with peers.

Session 7: Exiting Conversations: Strategies for politely ending a conversation.

Session 8: Managing Informal and Formal Gatherings: Skills for hosting and participating in both informal and formal social gatherings.

Session 9: Good Sportsmanship: Demonstrating appropriate behavior during games and competitive activities, including winning and losing gracefully.

Session 10: Managing Teasing and Embarrassing Situations: Developing coping strategies and composed responses

Session 11: Changing a Bad Reputation: Steps for improving a negative social impression over time through consistent, positive behavior.

Session 12: Handling Disagreements: Strategies for resolving disagreements calmly and respectfully.

Session 13: Addressing Rumors and Gossip: Techniques to respond to and dispel rumors.

Session 14: Graduation and Review: Consolidation of learned skills, celebration of achievements, and planning for future social maintenance.

Each session incorporated didactic instruction, therapist-facilitated role-playing of specific scenarios (e.g., initiating a conversation with a peer), and structured group discussions to solve common social problems (e.g., "How to respond if someone teases you?").

1.2 Interventionist Qualifications and Training

All SST group facilitators were licensed child psychiatrists or certified behavioral therapists with a minimum of one years of clinical experience in ASD intervention. Prior to the study, all facilitators completed a training program conducted by the principal investigator (a senior clinician with over a decade of SST experience). This training covered the detailed manual, role-playing facilitation techniques, behavior management strategies, and protocols for ensuring consistent delivery across all groups.

1.3 Consistency Control and Supervision

Rigorous measures were implemented to ensure intervention consistency and quality:

1) Standardized Manual: A detailed session-by-session manual was used by all facilitators.

2) Initial Training: As noted above.

3) Weekly Supervision: Facilitators attended weekly group supervision meetings with the principal investigator to review session progress, address challenges, and ensure strict adherence to the protocol.

4) Precise Monitoring: All sessions were audio-recorded. An independent rater, assessed these recordings using a self-designed behavior observation scale to score the classroom performance of each ASD. The maximum score being 5, the average score was 4.126. This system quantified adherence to the prescribed protocol.

2 Statistical analysis

Descriptive statistics were presented as mean ± standard deviation for continuous variables with normal distribution, and as frequency (percentage) for categorical variables. An independent samples t-test was employed for continuous variables following a normal distribution to compare baseline data between the training and control groups. A paired samples t-test was applied to assess pre- and post-training data within the training group, as well as baseline and endpoint data within the control group. The chi-square test was applied to categorical variables. The reduction rate = (the score before training - the score after training) / the score before training.

For the whole-brain analysis of the linear mixed model, a 10,000-permutation test (PT) (Eklund, Nichols, & Knutsson, 2016; Winkler, Ridgway, Douaud, Nichols, & Smith, 2016) was employed in conjunction with threshold-free cluster enhancement (TFCE) (Smith & Nichols, 2009). The analysis was conducted within an explicit mask comprising gray matter. Statistical inferences were made at the whole-brain level, with family-wise error (FWER) correction applied directly to the PT-TFCE output to control the false positive rate below 5%. This method offers superior reproducibility compared to traditional cluster-based thresholding (Chen, Lu, & Yan, 2018). The TFCE parameters were set to the default values (E=0.5, H=2). Brain regions exhibiting significant group × condition interaction were identified through calculations, and the average ReHo values of each subject in these regions were extracted.

3 Sensitivity Analysis Excluding Participants on Medication

The sensitivity analysis, which excluded 4 participants on psychotropic medication (2 from the training group and 2 from the control group), yielded results consistent with the primary analyses.

The linear mixed model analysis revealed significant group × condition interaction effects on the ReHo values within both the training and control groups. The pattern of significant Group × Condition interaction effects on ReHo within the five social brain regions (located in bilateral dorsolateral prefrontal cortex, the right medial frontal cortex, and the right insula) identified after PT-TFCE correction remained substantively unchanged.

Independent sample t-tests were administered to compare the baseline and endpoint scores of the ABC and SRS scales between the training and control groups. At baseline, the results revealed no significant differences in the total and factor scores of the ABC and SRS scales between the groups ($t = -1.512$ to 0.791, all $p > 0.05$). At the endpoint, the results showed that the training group had significantly lower scores in the irritability ((8.00 ± 7.81) vs. (12.56 ± 9.20); $t(32) = 5.712$, $p = 0.009$, Hedges' g = -0.531), hyperactivity factor ((9.56 ± 6.65) vs. (12.31 ± 10.08); $t(32) = 5.061$, $p = 0.024$, Hedges' g = -0.310), and total score of the ABC scale ((35.87 ± 22.65) vs. (45.19 ± 33.50); $t(32) = 4.230$, $p = 0.029$, Hedges' g = -0.308) compared to the control group, indicating statistically significant differences. No statistically significant differences were observed for the remaining factors of the ABC scale or any factors and the total score of the SRS scale ($t(32) = -2.813$ to 2.300, all $p > 0.05$).

A paired t-test was employed to compare the total and factor scores of the ABC and SRS scale between the training group's baseline and endpoint assessments. The analysis demonstrated that following 14 weeks of training, the total score of the ABC scale ((39.67 ± 28.92) vs. (32.50 ± 21.86); $t(17) = 2.416$, $p = 0.022$, Cohen's d = 0.277), the social withdrawal factor score ((12.77± 9.75) vs. (10.27 ± 7.84); $t(17) = 2.792$, $p = 0.009$, Cohen's d = 0.283), Stereotype behavior ((3.23±3.65) vs. (2.27± 2.83); $t(17) = 2.491$, $p = 0.019$, Cohen's d = 0.290), the total SRS score ((88.41±21.12) vs. (77.12± 18.12); $t(17) = 3.672$, $p = 0.001$, Cohen's d = 0.569) and the scores for social awareness ((9.18±2.52) vs. (8.18± 2.28); $t(17) = 2.831$, $p = 0.008$, Cohen's d = 0.711), social cognition ((18.27±4.69) vs. (16.18± 3.69); $t(17) = 3.197$, $p = 0.003$, Cohen's d = 0.494), social communication ((31.29±8.64) vs. (26.21± 7.83);$t(17) = 3.816$, $p = 0.001$), and social motivation ((14.12±4.86) vs. (11.91± 5.04); $t(17) = 4.046$, $p = 0.001$, Cohen's d = 0.619) were significantly reduced compared to baseline. However, no significant differences were found in the scores of the irritability, hyperactivity, and inappropriate speech factors of ABC and the autistic mannerisms factor of the SRS between baseline and endpoint assessments ($t(17) = 0.929$ to 1.706, all $p > 0.05$).

We also conducted independent sample t-tests to analyze the reduction rates from baseline to endpoint for each factor and the total score of the ABC and SRS scale between the training and control groups. The results revealed that the reduction rate of the social awareness factor in the training group was significantly higher than that in the control group [training group vs. control group: (0.19 ± 0.21) vs. (0.00 ± 0.15); t (32) = 3.027, p = 0.006, Hedges' g = 1.008]. No significant differences were observed in the reduction rates for the remaining factors or the total SRS and ABC score between the two groups (p > 0.05).

Linear regression was employed to explore the correlation between alterations in ReHo and changes in symptom severity pre- and post-training within the training group. No significant linear relationships were observed between the variations in the changes in ABC and SRS scores pre- and post-training and the baseline-to-endpoint ReHo value differential (all p>0.01 after Bonferroni correction).

4 Supplementary Tables

For more information on Supplementary Material and for details on the different file types accepted, please see here.

Supplementary Table 1 Comorbidity and medication in the training group and control group

Subjects	Comorbidity	Medication（Daily dosage）
Subject 1 in training group	ADHD	Aripiprazole（10mg/d）
Subject 2 in training group	Recurrent depressive disorder	Venlafaxine（75mg/d），aripiprazole（15mg/d），escitalopram oxalate（10mg/d）
Subject 1 in control group	Suspicious ADHD，childhood emotion disorder	Sertraline（50mg/d）
Subject 2 in control group	Childhood emotion disorder	Aripiprazole（5mg/d）

Note: ADHD, Attention-Deficit / Hyperactivity Disorder

Supplementary Table 2. Behavioral outcomes (ABC and SRS): descriptive data and statistical comparisons

A. Descriptive Data: Mean Scores at Baseline and Endpoint

Scale/Subscale	Training Group (n = 20)		Control Group (n = 18)	
	Baseline	Endpoint	Baseline	Endpoint
ABC Scale				
Irritability	6.35 ± 6.77	4.65 ± 4.43	11.88 ± 9.28	11.00 ± 7.45
Social withdrawal	11.29 ± 8.77	8.21 ± 6.27	13.82 ± 10.35	12.47 ± 8.49
Stereotypic behavior	2.82 ± 2.81	2.18 ± 1.91	3.18 ± 4.16	2.24 ± 3.35
Hyperactivity	9.18 ± 6.54	6.53 ± 3.45	11.82 ± 10.17	11.24 ± 7.55
Inappropriate speech	2.65 ± 1.69	2.18 ± 1.98	3.00 ± 2.76	2.47 ± 2.03
The total score of ABC	32.29 ± 20.52	23.65 ± 11.19	43.71 ± 33.37	39.41 ± 25.47
SRS Scale				
Social awareness	11.10 ± 2.85	9.15 ± 2.64	10.44 ± 2.73	10.50 ± 2.18
Social cognition	18.25 ± 4.52	15.60 ± 3.98	17.39 ± 4.98	16.39 ± 3.13
Social communication	31.75 ± 9.23	25.85 ± 7.09	30.17 ± 7.52	26.94 ± 8.29
Social motivation	13.15 ± 5.19	11.05 ± 5.42	15.00 ± 4.65	13.72 ± 4.84

Scale/Subscale	Training Group (n = 20)		Control Group (n = 18)	
	Baseline	Endpoint	Baseline	Endpoint
Autistic mannerisms	15.20 ± 6.89	14.30 ± 4.41	14.29 ± 5.19	14.83 ± 4.49
The total score of SRS	89.45 ± 21.62	75.95 ± 16.89	87.28 ± 21.18	82.39 ± 18.31

B. Statistical Comparisons (Effect sizes with 95% Confidence Intervals)

B1. Within-Training Group Comparisons (Paired t-tests)

Scale/Subscale	*t* (19)	*p*	Cohen's d [95% CI]
ABC			
Irritability	1.404	0.179	0.292 [-0.153, 0.737]
Social withdrawal	2.806	0.013*	0.626 [0.097, 1.156]
Stereotypic behavior	1.079	0.297	0.258 [-0.252, 0.767]
Hyperactivity	2.111	0.051	0.513 [-0.012, 1.038]
Inappropriate speech	1.000	0.332	0.259 [-0.284, 0.803]
The total score of ABC	2.329	0.033*	0.501 [-0.048, 1.050]

Scale/Subscale	*t* (19)	*p*	Cohen's d [95% CI]
SRS			
Social awareness	3.577	0.002**	0.703 [0.190, 1.216]
Social cognition	2.950	0.008**	0.618 [0.087, 1.148]
Social communication	2.933	0.009**	0.716 [0.143, 1.288]
Social motivation	2.333	0.031*	0.395 [-0.068, 0.858]
Autistic mannerisms	0.595	0.559	0.153 [-0.407, 0.714]
The total score of SRS	2.756	0.013*	0.690 [0.111, 1.268]

B2. Within-Control Group Comparisons (Paired t-tests)

Scale/Subscale	*t* (17)	*p*	Cohen's d [95% CI]
ABC			
Irritability	0.776	0.449	0.104 [-0.179, 0.388]
Social withdrawal	1.203	0.249	0.140 [-0.115, 0.395]
Stereotypic behavior	2.426	0.027*	0.253 [-0.054, 0.560]

Scale/Subscale	*t* (17)	*p*	Cohen's d [95% CI]
Hyperactivity	0.421	0.679	0.063 [-0.268, 0.395]
Inappropriate speech	0.824	0.422	0.215 [-0.366, 0.795]
The total score of ABC	1.132	0.274	0.142 [-0.137, 0.422]
SRS			
Social awareness	-0.142	0.889	-0.024 [-0.406, 0.358]
Social cognition	1.290	0.214	0.239 [-0.171, 0.649]
Social communication	2.631	0.018*	0.416 [0.011, 0.821]
Social motivation	2.135	0.048*	0.269 [-0.050, 0.588]
Autistic mannerisms	-0.491	0.630	-0.111 [-0.627, 0.405]
The total score of SRS	1.644	0.119	0.247 [-0.084, 0.577]

B3. Between-Group Comparisons at Baseline (Independent t-tests)

Scale/Subscale	*t* (36)	*p*	Hedges' g [95% CI]
ABC			

Scale/Subscale	*t* (36)	*p*	Hedges' g [95% CI]
Irritability	-1.636	0.111	-0.666 [-1.498, 0.166]
Social withdrawal	-0.348	0.730	-0.258 [-1.732, 1.217]
Stereotypic behavior	-0.096	0.924	-0.098 [-2.209, 2.013]
Hyperactivity	-0.944	0.352	-0.302 [-0.944, 0.341]
Inappropriate speech	-0.509	0.614	-0.151 [-0.740, 0.438]
The total score of ABC	-0.958	0.345	-0.395 [-1.222, 0.433]
SRS			
Social awareness	0.723	0.474	0.236 [-0.420, 0.891]
Social cognition	0.559	0.861	0.182 [-1.933, 2.297]
Social communication	0.576	0.568	0.188 [-0.470, 0.846]
Social motivation	-1.151	0.257	-0.372 [-1.022, 0.278]
Autistic mannerisms	0.462	0.647	0.145 [-0.481, 0.771]
The total score of SRS	0.313	0.756	0.101 [-0.557, 0.760]

B4. Between-Group Comparisons at Endpoint (Independent t-tests)

Scale/Subscale	*t* (36)	p	Hedges' g [95% CI]
ABC			
Irritability	5.615	0.005**	-1.104 [-1.724, -0.485]
Social withdrawal	1.129	0.099	-0.556 [-1.219, 0.108]
Stereotypic behavior	2.317	0.950	-0.023 [-0.797, 0.750]
Hyperactivity	5.270	0.026*	-0.819 [-1.461, -0.177]
Inappropriate speech	0.017	0.672	-0.144 [-0.809, 0.521]
The total score of ABC	4.689	0.026*	-0.805 [-1.443, -0.166]
SRS			
Social awareness	3.277	0.092	-0.551 [-1.197, 0.095]
Social cognition	1.842	0.505	-0.218 [-0.864, 0.428]
Social communication	0.062	0.663	-0.142 [-0.784, 0.500]
Social motivation	0.279	0.119	-0.515 [-1.170, 0.140]

Scale/Subscale	*t* (36)	p	Hedges' g [95% CI]
Autistic mannerisms	0.074	0.714	-0.119 [-0.762, 0.524]
The total score of SRS	0.006	0.269	-0.365 [-1.018, 0.288]

Note: ABC: Aberrant Behavior Checklist; SRS: Social Responsiveness Scale. Data are presented as mean ± SD. For within-group comparisons (paired t-tests), effect sizes are reported as Cohen's d with 95% confidence intervals (CI). For between-group comparisons (independent t-tests), effect sizes are reported as Hedges' g with 95% CI. * $p<0.05$； ** $p<0.01$

4 References

Chen, X., Lu, B., & Yan, C. G. (2018). Reproducibility of R-fMRI metrics on the impact of different strategies for multiple comparison correction and sample sizes. Hum Brain Mapp, 39(1), 300-318. doi:10.1002/hbm.23843

Eklund, A., Nichols, T. E., & Knutsson, H. (2016). Cluster failure: Why fMRI inferences for spatial extent have inflated false-positive rates. Proc Natl Acad Sci U S A, 113(33), E4929. doi:10.1073/pnas.1612033113

Smith, S. M., & Nichols, T. E. (2009). Threshold-free cluster enhancement: addressing problems of smoothing, threshold dependence and localisation in cluster inference. Neuroimage, 44(1), 83-98. doi:10.1016/j.neuroimage.2008.03.061

Winkler, A. M., Ridgway, G. R., Douaud, G., Nichols, T. E., & Smith, S. M. (2016). Faster permutation inference in brain imaging. Neuroimage, 141, 502-516. doi:10.1016/j.neuroimage.2016.05.068
